# Different timing and risk factors of cause-specific pancreas graft loss after simultaneous pancreas kidney transplantation

**DOI:** 10.1038/s41598-022-22321-x

**Published:** 2022-10-21

**Authors:** Yoshito Tomimaru, Shogo Kobayashi, Toshinori Ito, Kazuki Sasaki, Yoshifumi Iwagami, Daisaku Yamada, Takehiro Noda, Hidenori Takahashi, Takashi Kenmochi, Yuichiro Doki, Hidetoshi Eguchi

**Affiliations:** 1 Department of Gastroenterological Surgery, Graduate School of Medicine, Osaka University, 2-2 Yamadaoka E-2, Suita, Osaka 565-0871 Japan; 2 The Japan Pancreas Transplant Registry, The Japanese Pancreas and Islet Transplant Association, Suita, Japan; 3 Osaka Center for Cancer and Cardiovascular Disease Prevention, Osaka, Japan; 4 Department of Transplantation and Regenerative Medicine, School of Medicine, Fujita Health University, Toyoake, Japan

**Keywords:** Endocrine system and metabolic diseases, Gastroenterology

## Abstract

In cases after pancreas transplantation (PTx), the pancreas graft might be lost for various reasons, including a pancreatoduodenal graft-related complication or patient death with a functioning graft. Although the causes seem to have distinct characteristics, the causes of pancreas graft loss have not been characterized. This study aimed to characterize the causes of pancreas graft loss by analyzing data from a Japanese nationwide registry. This study included 391 patients who received simultaneous pancreas-kidney transplantation among 461 patients with PTx during the study period in approved institutions in Japan. We characterized each cause in terms of the timing of the cause-specific graft loss and preoperative factors associated with graft loss based on nationwide data from a Japanese nationwide registry. Among the 391 cases, 113 lost the pancreas graft due to patient death with a functioning graft (DWFG; n = 34, 44.2%), graft thrombus (n = 22, 28.6%), or chronic rejection (CR; n = 7, 9.1%). Average (± standard deviation) time from PTx to graft loss due to DWFG, graft thrombus, and CR was 3.70 ± 4.36, 0.02 ± 0.01, and 2.37 ± 2.08 years, respectively. Duration of type 1 diabetes mellitus and donor body mass index were significantly associated with pancreas graft loss due to DWFG and graft thrombus, respectively. This characterization showed that the timing and preoperative factors associated with pancreas graft loss were significantly different for different causes of graft loss. These results may inform PTx follow-up protocols to ensure that appropriate care is based on the cause of graft loss.

## Introduction

Pancreas transplantation (PTx), which was first performed to treat type 1 diabetes mellitus in 1966, is an established therapeutic procedure for patients with diabetes mellitus^1–3^. When successfully performed, PTx can restore endogenous insulin secretion, which results in normal glucose metabolism in patients with diabetes. Due to the therapeutic effects of insulin on glucose metabolism, PTx can reduce diabetes-related complications, such as retinopathy, nephropathy, neuropathy, gastroparesis, and cardiovascular diseases. Furthermore, considering that the complications can lead to impaired quality of life and the occurrence of life-threatening events, the clinical benefit of PTx is evident for patients with diabetes mellitus^4–8^. However, the outcomes of PTx remain unsatisfactory. For example, in Japan, the 5- and 10-year survival rates for post-transplant pancreas grafts are 76.2% and 67.4%, respectively^9,10^. These post-transplant survival rates are comparable to outcomes reported in other countries^11^, and this universal problem of unsatisfactory PTx outcomes is thought to be due to post-transplant pancreas graft loss. Several causes of pancreas graft loss have been reported, including pancreatoduodenal graft-related complications and death with a functioning graft (DWFG). The complications include a graft thrombus, duodenal graft perforation, immunological rejection, and recurrence of type 1 diabetes mellitus. DWFG can be caused by cerebrovascular disease, cardiovascular disease, infection, or malignancy. Thus, pancreas graft loss has a variety of causes^9,10^. Each cause is likely to exhibit different clinical characteristics, including the frequency of the cause, the timing of the occurrence, and influencing factors. To the best of our knowledge, this is the first study to describe pancreas graft loss by etiology and clinical characteristics. We hope that investigations of the clinical characteristics influencing pancreas allograft failure may enhance our understanding of pancreas graft loss and improve management and outcomes. Thus, the present study aimed to characterize the specific causes of pancreas graft loss after PTx, including predictive preoperative factors for cause-specific pancreas graft loss based on data from a Japanese nationwide registry.

## Materials and methods

### Patients

This study included 461 recipients of PTx from a deceased donor after brain death (DBD) or a deceased donor after circulatory death (DCD). The 461 procedures were performed between 2000 and 2021 to treat type 1 diabetes mellitus at 19 institutions approved by the Japanese Pancreas and Islet Transplant Association. For the present study, we extracted anonymized clinical data for the 461 cases from the Japan Pancreas Transplant Registry of the Japanese Pancreas and Islet Transplant Association. Among the 461 PTx cases, including 458 from DBD and 3 from DCD, 391 underwent simultaneous pancreas-kidney transplantation (SPK), 51 received a pancreas after kidney transplantation (PAK), and 19 underwent PTx alone (PTA). To simplify the study model, the 391 cases with SPK, the predominant category of PTx, were included in this study. Clinical data were analyzed to identify perioperative factors and post-transplant outcomes.

### Assessment and definition

Pancreas graft loss was defined as a return of serum C-peptide to < 0.3 ng/ml. Kidney graft loss was defined as a return to dialysis. In defining graft loss, DWFG was considered a graft failure. Expanded donor criteria were defined based on criteria reported by Kapur et al. and Troppmann et al.^12,13^.

### Ethics and statistical analysis

Given the anonymization of the data, the study was exempt from approval by the Institutional Review Board of Osaka University Hospital based on the ethical guideline in Japan, "Ethical Guidelines for Medical and Biological Research Involving Human Subjects." Informed consent was waived for the same reason. This study was conducted in accordance with the Declaration of Helsinki. Measured data are described as the mean ± standard deviation for continuous variables, and as the number (%) for categorical variables. Differences between groups were assessed by chi-squared, Fisher’s exact test, or Mann–Whitney U test, as appropriate. The cumulative incidence of pancreas graft loss was assessed by the Kaplan–Meier method. Hazard rates were measured in terms of the number of events per the number of patients at risk over a 1-year interval. To visualize the hazard rate pattern, we created smoothed curves with a Kernel smoothing procedure. Univariate and multivariate analyses of Cox proportional hazards regression models were performed to identify preoperative factors that may affect pancreas graft loss. Multivariate analysis was performed using significant factors and those with a tendency toward significance in the univariate analyses. Statistical analyses were performed using JMP Pro 14 software (SAS Institute Inc., Cary, NC, USA). p-values < 0.05 were considered significant, and p-values < 0.1 were considered to trend towards significance.

## Results

### Incidence and causes of pancreas graft loss after PTx

Post-transplant pancreas graft loss was identified in 77 of the 391 SPK cases (19.7%). The cumulative incidence of pancreas graft loss 1, 3, 5, and 10 years after PTx was 12.5%, 15.2%, 17.2%, and 24.9%, respectively. The pancreas graft loss was caused by DWFG (n = 34, 44.2%), graft thrombus (n = 22, 28.6%), chronic rejection (CR; n = 7, 9.1%), duodenal graft perforation (n = 6, 7.8%), acute rejection (AR; n = 3, 3.9%), recurrence of type 1 diabetes mellitus (n = 3, 3.9%), and pancreatoduodenal graft-related complications other than graft thrombus or duodenal graft perforation (n = 2, 2.6%). DWFG was caused by infection (n = 13), cerebro-cardiac disease (n = 10), malignancy (n = 4), multiple organ failure (n = 3), renal deficiency (n = 1), graft versus host disease (n = 1), and unknown reasons (n = 2). These causes are summarized in Table 1.

**Table 1:** Cause of pancreas graft loss including cause of the DWFG in cases after PTx.

Cause	n (%)
**DWFG**	34 (44.2%)
Infection	13 (38.2%^a^)
Cerebro-cardiac disease	10 (29.4%^a^)
Malignancy	4 (11.8%^a^)
Multiple organ failure	3 (8.8%^a^)
Renal deficiency	1 (2.9%^a^)
Graft versus host disease	1 (2.9%^a^)
Unknown reason	2 (5.9%^a^)
Graft thrombus	22 (28.6%)
CR	7 (9.1%)
Duodenal graft perforation	6 (7.8%)
AR	3 (3.9%)
Recurrence of type 1 diabetes mellitus	3 (3.9%)
Pancreatoduodenal graft-related complications other than graft thrombus or duodenal graft perforation	2 (2.6%)

*AR* acute rejection, *CR* chronic rejection, *DWFG* death with a functioning graft, *PTx* pancreas transplantation.

^a^Percentage of all DWFG cases.

### Clinical background based on cause of pancreas graft loss

In the present study, we focused on the three major causes of post-PTx pancreas graft loss: DWFG, graft thrombus, and CR. First, we compared the clinical backgrounds of these three groups (Table 2). In regard to donor-related factors (age, sex distribution, proportions of deaths caused by cerebrovascular accident, hemodynamic stability, cardiopulmonary arrest episodes, and marginal donors as defined by Kapur et al. and Troppmann et al.^12,13^), there were no significant differences among the groups. In contrast, donor body mass index (BMI) was significantly higher in the graft thrombus group than the DWFG group (p = 0.0279). The number of human leukocyte antigen mismatches was comparable among the groups. With regard to recipient-related factors, sex distribution, the duration of type 1 diabetes mellitus, the proportion of patients that required dialysis, the duration of dialysis, and the time after the registration of PTx were also not significantly different among the groups, whereas recipient age tended to be older in the DWFG group than the CR group (p = 0.058) and recipient BMI tended to be higher in the graft thrombus group than the DWFG group (p = 0.0615). There were also no significant differences in PTx-related factors, such as the duration of pancreas or kidney graft ischemia, among the three groups.

**Table 2:** Clinical characteristics of patients who lost pancreas grafts due to three major causes.

Factor	DWFG (n = 34)	Graft thrombus (n = 22)	CR (n = 7)	p-value
**Donor-related factors**				
Age, years	39 ± 15	45 ± 12	44 ± 19	
Sex (male)	20 (58.8%)	14 (63.6%)	5 (71.4%)	
BMI, kg/m^2^	21.8 ± 3.2	23.7 ± 3.1	22.4 ± 4.1	0.0279 (DWFG vs. graft thrombus)
Cause of death (CVA)	17 (50.0%)	15 (68.2%)	4 (57.1%)	
Hemodynamic stability (+)	18 (52.9%)	9 (40.9%)	4 (57.1%)	
Cardiopulmonary arrest (+)	15 (44.1%)	11 (50.0%)	2 (28.6%)	
Marginality by Kapur’s criteria (+)	23 (67.6%)	19 (86.4%)	6 (85.7%)	
Marginality by Troppmann’s criteria (+)	21 (61.8%)	17 (77.3%)	5 (71.4%)	
HLA mismatch number	2 ± 1	3 ± 1	2 ± 1	
**Recipient-related factors**				
Age, years	48 ± 9	45 ± 8	41 ± 9	0.058 (DWFG vs. CR)
Sex (male)	11 (32.4%)	8 (36.4%)	3 (42.9%)	
BMI, kg/m^2^	20.4 ± 2.8	22.1 ± 3.9	21.4 ± 2.3	0.0615 (DWFG vs. graft thrombus)
Duration of type 1 diabetes mellitus, years	32.8 ± 9.4	31.0 ± 10.3	28.9 ± 12.6	
Dialysis (+)	33 (97.1%)	21 (95.5%)	7 (100.0%)	
Duration of dialysis, years	9.6 ± 6.9	8.9 ± 7.1	6.0 ± 6.2	
Duration after registration, years	4.6 ± 4.3	4.0 ± 3.0	2.6 ± 3.1	
**PTx-related factors**				
Ischemic time of pancreas graft, min	784 ± 181	843 ± 141	764 ± 297	
Ischemic time of kidney graft, min	671 ± 181	636 ± 185	670 ± 296	

Data are expressed as the number of patients (%) or the mean ± standard deviation. The differences between groups were assessed by chi-squared, Fisher’s exact test, or Mann–Whitney U test, as appropriate. p-values are shown only in the comparisons with p < 0.1

*BMI* body mass index, *CR* chronic rejection, *CVA* cerebrovascular accident, *DWFG* death with a functioning graft, *HLA* human leukocyte antigen, *PTx* pancreas transplantation.

### Timing of cause-specific pancreas graft loss

**Figure 1:**
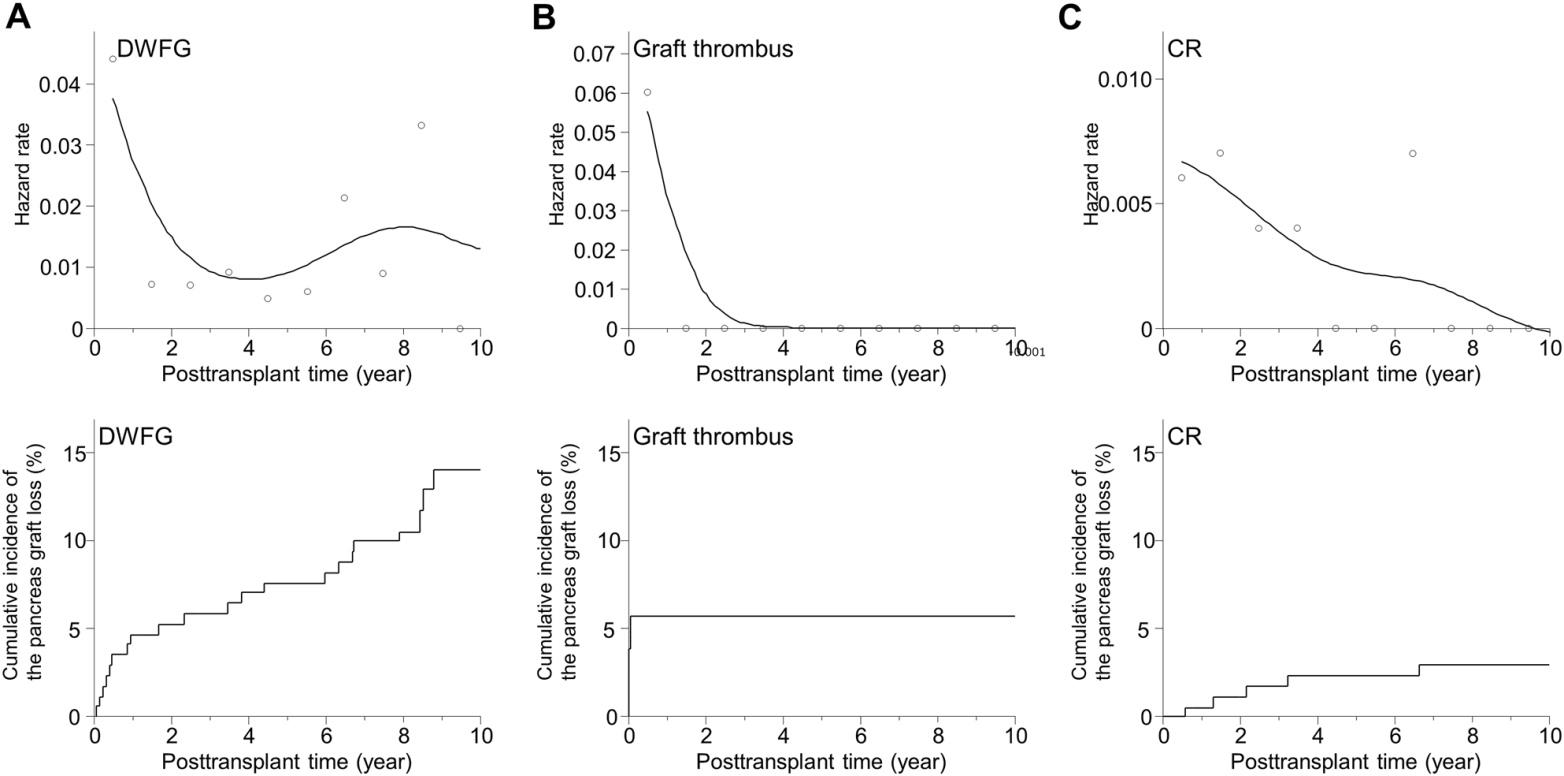
Hazard rates and cumulative incidences of specific causes of pancreas graft loss in SPK cases. Upper panels: Hazard rates for graft loss within 10 years due to (**A**) DWFG, (**B**) graft thrombus, and (**C**) CR. Solid lines show smoothed curves obtained with a Kernel smoothing procedure based on the causes of the pancreas graft loss. The hazard rates were measured in terms of the number of events per the number of patients at risk over a 1-year interval. The smoothed curves were created to visualize the pattern of the hazard rates with a Kernel smoothing procedure. Lower panels: Cumulative incidences of post-transplant pancreas graft loss due to (**A**) DWFG, (**B**) graft thrombus, and (**C**) CR (solid lines). The cumulative incidence was assessed by the Kaplan–Meier method. *CR* chronic rejection, *DWFG* death with a functioning graft, *SPK* simultaneous pancreas-kidney transplantation.

**Figure 2:**
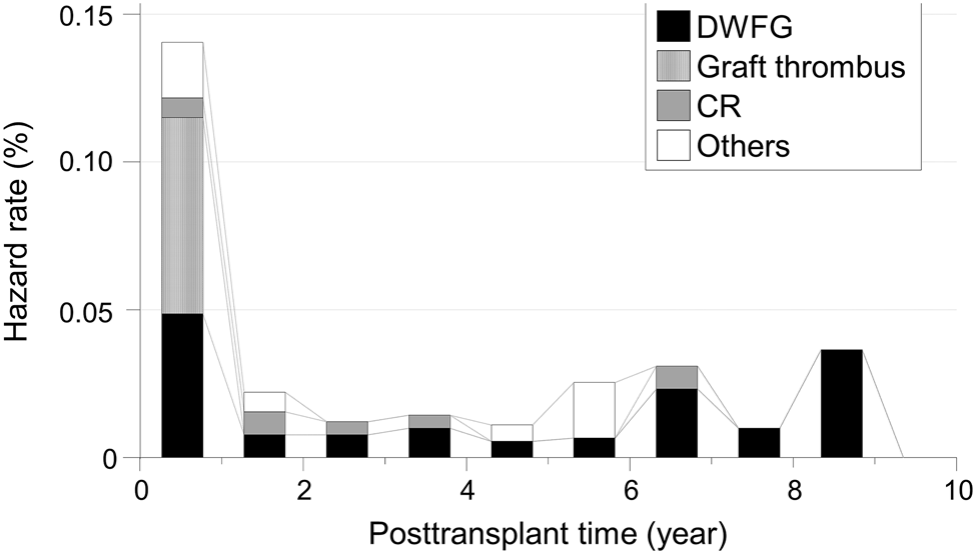
Whole hazard rate of pancreas graft loss within 10 years with its cause in SPK cases. *CR* chronic rejection, *DWFG* death with a functioning graft, *SPK* simultaneous pancreas-kidney transplantation.

**Figure 3:**
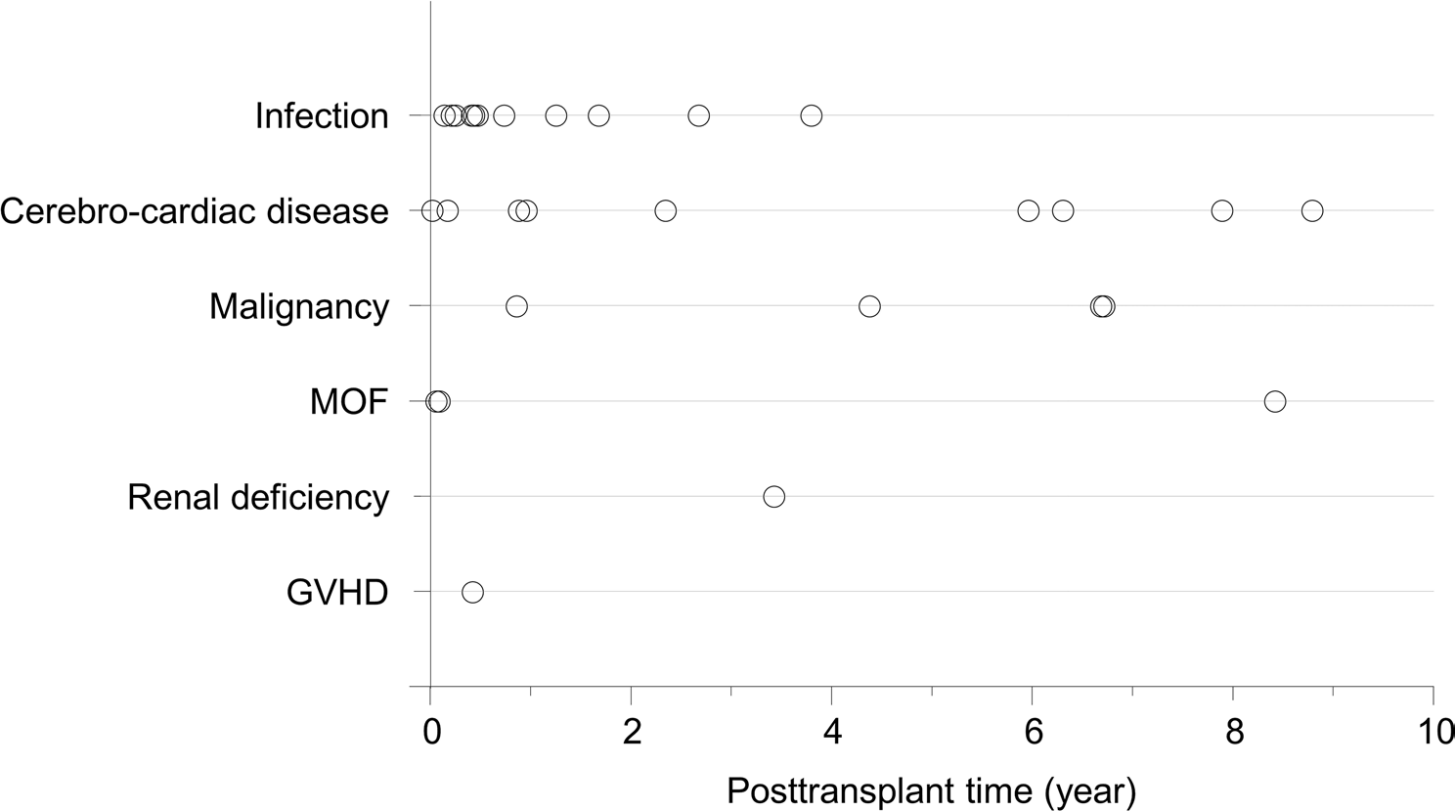
Timing of pancreas graft loss due to DWFG based on the cause of DWFG in SPK cases. ACH plot indicates the timing and cause of DWFG. *DWFG* death with a functioning graft, *GVHD* graft versus host disease, *MOF* multiple organ failure, *SPK* simultaneous pancreas-kidney transplantation.

Next, we investigated the timing of graft loss due to the three major causes (Table 3). The average and median time from SPK to graft loss due to DWFG was 3.70 and 1.47 years [range 0.02 years (0.86 weeks) to 17.97 years], respectively. The 90th and 95th percentiles were 8.71 and 11.20 years, respectively. The loss occurred within the first postoperative month in 5.9%, within the first year in 47.1%, and within 5 years in 67.6%. In contrast, the average and median time from PTx to pancreas graft loss due to graft thrombus was 0.02 (0.86 weeks) and 0.01 years [0.57 weeks) (range 0.00 years (0.14 weeks) to 0.44 years (2.29 weeks)], respectively. The 90th and 95th percentiles were 0.40 years (2.00 weeks) and 0.40 years (2.29 weeks), respectively, and the loss occurred within the first postoperative month in 100.0%. The average and median time from PTx to the graft loss due to CR was 2.37 and 2.08 years [range 0.58 years (30.14 weeks) to 6.63 years], respectively. The 90th and 95th percentiles were 4.58 and 5.61 years, respectively. Though no cases developed CR within the first postoperative month, the loss occurred within the first year in 28.6% and within 5 years in 42.9%. Thus, the timing of pancreas graft loss was significantly different among the three causes. Notably, the time to graft loss was significantly shorter in the graft thrombus group than in the DWFG group and CR group (p = 0.0002 and p < 0.0001, respectively). The grafts in the DWFG group lasted longer than the grafts in the other groups. Although 47.1% were lost within the first year, 33.4% survived longer than 5 years, and two cases developed DWFG after more than 10 years. In the CR group, approximately 70.0% of the grafts lasted more than 1 year. The differences in the timing of pancreas graft loss were reflected in differences in the hazard rates of the three specific causes and the cumulative incidence of pancreas graft loss in the Kaplan–Meier curves (Fig. 1). Figure 2 shows the whole hazard rate of pancreas graft loss per year with the causes based on the timing. Thus, the timing of pancreas graft loss was significantly different among the causes. We also investigated the timing of DWFG-derived pancreas graft loss. The timing also varied among the causes of DWFG (Fig. 3).

**Table 3:** Timing of pancreas graft loss after a pancreas transplant based on cause.

Factor	DWFG (n = 34)	Graft thrombus (n = 22)	CR (n = 7)
**Time to graft loss, years**			
Average ± SD	3.70 ± 4.36	0.02 ± 0.01	2.37 ± 2.08
Median (range)	1.47 (0.02–17.97)	0.01 (0.00–0.44)	2.08 (0.58–6.63)
90th percentile	8.70	0.04	4.58
95th percentile	11.20	0.40	5.61
Within first month, %	5.9	100.0	0
Within first year, %	47.1	100.0	28.6
Within first 5 years, %	67.6	100.0	42.9
Within first 10 years, %	91.2	100.0	100.0

The time to graft loss was significantly shorter in the graft thrombus group than in the DWFG group and CR group (p = 0.0002 and p < 0.0001, respectively). There was no significant difference between the DWFG group and CR group (p = 0.4394). The differences between groups were assessed by the Mann–Whitney U test.

*CR* chronic rejection, *DWFG* death with a functioning graft, *SD* standard deviation.

### Preoperative factors affecting cause-specific pancreas graft loss

Based on the results in the three groups, we investigated potential factors that might affect pancreas graft loss in univariate and multivariate Cox proportional hazards regression analyses. Preoperative factors associated with pancreas graft loss were significantly different among the three groups. The univariate analysis showed that, in the DWFG group, pancreas graft loss was significantly associated with recipient age [hazard ratio (HR) = 1.075, p = 0.0007], the duration of type 1 diabetes mellitus in the recipient (hazard ratio = 1.090, p = 0.0001), the duration of dialysis in the recipient (HR = 1.104, p = 0.0010), and the duration after the registration for PTx (HR = 1.102, p = 0.0399; Table 4). In the graft thrombus group, the univariate analysis showed that the donor BMI was significantly associated with pancreas graft loss (HR = 1.147, p = 0.0144). The recipient BMI tended to be associated (HR = 1.119, p = 0.0909). In the CR group, no preoperative factors were significantly associated with pancreas graft loss in the univariate analysis.

**Table 4:** Cox proportional hazards regression p-values from univariate analyses of preoperative factors potentially influencing pancreas graft loss after PTx according to specific cause.

Factor	DWFG	Graft thrombus	CR
**Donor-related factors**			
Age (years)	0.6741	0.1486	0.5047
Sex (male/female)	0.3994	0.4160	0.3585
BMI (kg/m^2^)	0.6720	0.0144	0.7473
Cause of death (CVA/other)	0.9791	0.1641	0.6172
Hemodynamic stability (−/ +)	0.4757	0.3097	0.6931
Cardiopulmonary arrest (−/ +)	0.8955	0.6966	0.3671
Marginality by Kapur’s criteria (−/ +)	0.6171	0.1081	0.3807
Marginality by Troppmann’s criteria (−/ +)	0.9269	0.1574	0.5636
HLA mismatch number	0.2236	0.9256	0.1018
**Recipient-related factors**			
Age (years)	0.0007	0.8495	0.2869
Sex (male/female)	0.3908	0.7411	0.8409
BMI (kg/m^2^)	0.3460	0.0909	0.5928
Duration of type 1 diabetes mellitus (years)	0.0001	0.2450	0.8417
Dialysis (−/ +)	0.8521	0.6380	–
Duration of dialysis (years)	0.0010	0.1224	0.7327
Duration after registration (years)	0.0399	0.4338	0.4579

*BMI* body mass index, *CR* chronic rejection, *CVA* cerebrovascular accident, *DWFG* death with a functioning graft, *HLA* human leukocyte antigen, *PTx* pancreas transplantation.

**Figure 4:**
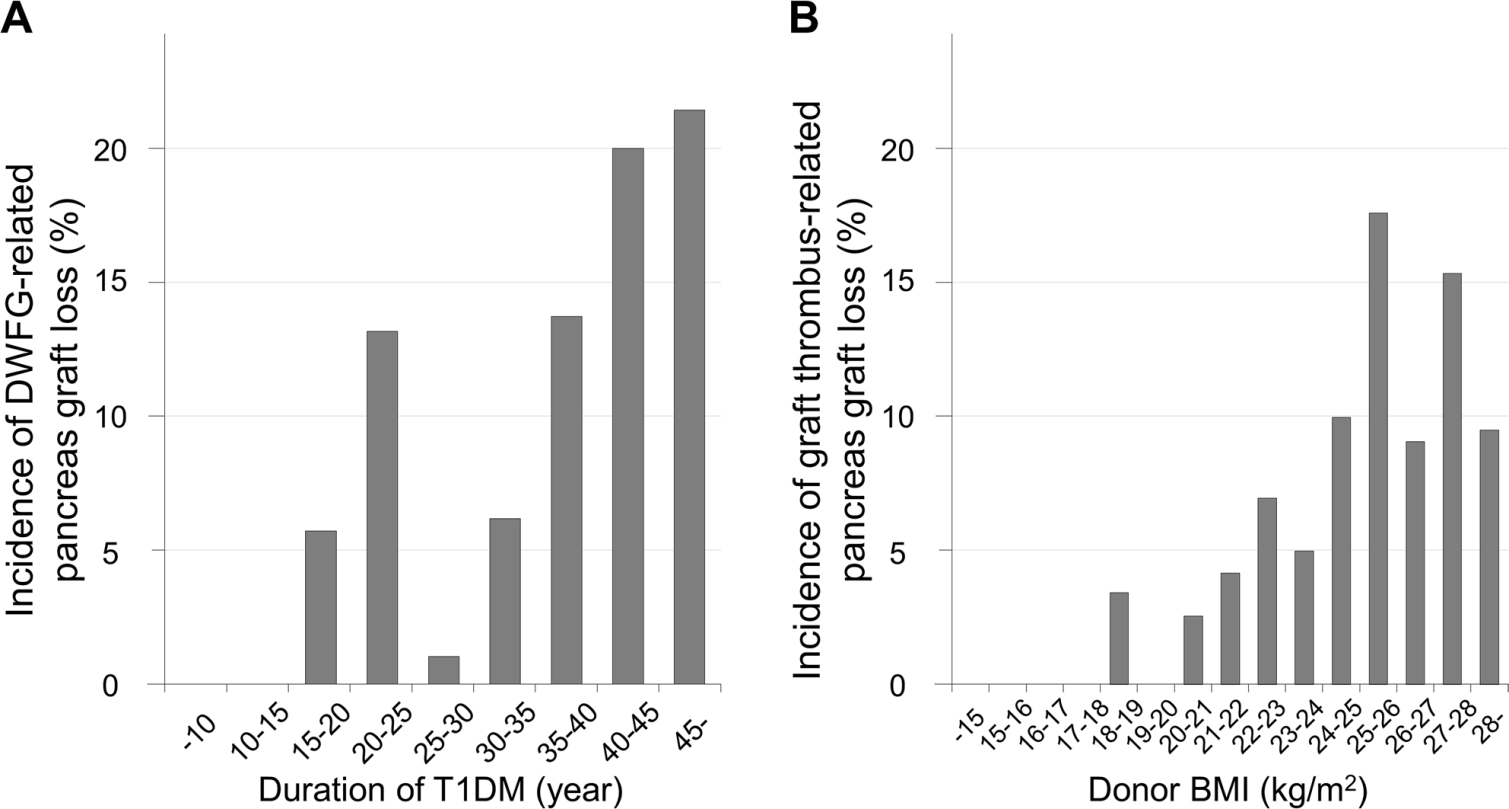
Incidence of pancreas graft loss due to each cause based on the independent factor associated with the graft loss. (**A**) Incidence of DWFG-derived pancreas graft loss based on the duration of type 1 diabetes mellitus. (**B**) Incidence of graft thrombus-derived pancreas graft loss based on donor BMI. *BMI* Body mass index, *DWFG* death with a functioning graft.

Finally, to identify independent preoperative factors significantly associated with pancreas graft loss, a multivariate analysis was performed using the significant factors and those with a tendency toward significance in the subgroups based on the causes of pancreas graft loss (Table 5). The analysis demonstrated that the duration of type 1 diabetes mellitus in the recipient was the only factor associated with DWFG-derived pancreas graft loss (HR = 1.064, p = 0.0276). As shown in Fig. 4A. The incidence of DWFG-derived pancreas graft loss was increased with the duration of type 1 diabetes mellitus. When the patients were divided into two groups based on the average (29.1 years) considering easy clinical application, a more than average duration was significantly associated with DWFG-related pancreas graft loss compared to less than average duration (HR = 2.458, p = 0.0134). In the graft thrombus group, donor BMI was the only factor that remained significantly associated with pancreas graft loss (HR = 1.147, p = 0.0160). The incidence of pancreas graft loss due to graft thrombus was increased based on the donor BMI (Fig. 4B). When the patients were divided into two groups based on the average (21.9 kg/m^2^), a more than average BMI was significantly associated with graft thrombus-related pancreas graft loss compared to less than average BMI (HR = 2.991, p = 0.0221). Thus, the significant preoperative factors independently associated with pancreas graft loss were different between the DWFG group and the graft thrombus group, and no preoperative factors were significantly associated with pancreas graft loss in the CR group.

**Table 5:** Multivariate analysis of preoperative factors potentially influencing pancreas graft loss due to DWFG or graft thrombus after PTx.

Factor	DWFG			Graft thrombus		
	HR	95% CI	p-value	HR	95% CI	p-value
**Donor-related factors**						
BMI (kg/m^2^)				1.147	1.026–1.282	0.0160
**Recipient-related factors**						
Age (years)	1.040	0.986–1.096	0.1527			
BMI (kg/m^2^)				1.114	0.977–1.271	0.1057
Duration of type 1 diabetes mellitus (years)	1.064	1.007–1.124	0.0276			
Duration of dialysis (years)	1.033	0.962–1.110	0.3646			
Duration after registration (years)	1.021	0.915–1.139	0.7142			

Cox proportional hazards regression used significant factors from the univariate analysis.

*DWFG* death with a functioning graft, *HR* hazard ratio, *PTx* pancreas transplantation, *CI* confidence interval.

## Discussion

In the present study, we characterized three major specific causes of pancreas graft loss after SPK based on the nationwide PTx registry in Japan. The three causes were selected based on the frequency of occurrence in anticipation of the potential clinical applications of our results. We found that the timing of pancreas graft loss and associated preoperative factors were remarkably different for the three causes in the SPK cases.

Previous studies have discussed factors that significantly influence PTx outcomes in patients with type 1 diabetes mellitus, such as the ages of the recipient and donor, PTx category, pancreas graft preservation time, and immunosuppressive regimen^14–18^. However, these analyses included the entire PTx cohort without focusing on the causes of graft loss, even including PAK and PTA cases with considerably varied characteristics. Considering the potentially different characteristics of the causes, some significant factors associated with prognosis may have been masked in previous studies that included an entire population. Furthermore, due to the pooling of the various causes of pancreas graft loss, the statistical analyses may have overestimated the significance of certain factors. Ito et al. demonstrated that no factors significantly influenced pancreatic graft survival in a multivariate analysis of post-PTx outcomes based on the Japanese database^19^. If they had performed the analysis based on cause-specific subgroups, their results would have been similar to those reported in the present study.

The present study focused on the three major causes of pancreas graft loss and provided several new findings in the SPK cases. First, in the DWFG group, the timing of graft loss was not limited to a certain period, but varied widely. Moreover, the duration of type 1 diabetes mellitus was the only factor associated with graft loss due to DWFG. To date, no studies have investigated DWFG in post-PTx cases; however, considering the nature of DWFG, and particularly the causes of DWFG, our results were considered realistic. On the other hand, the predominant timing of each cause of DWFG may be different; in other words, infection-derived DWFG may develop early after PTx, whereas DWFG due to malignancy may occur long time after PTx (Fig. 3). In contrast, the timing of the development of cerebro-cardiac disease-derived DWFG seems to vary widely. Further investigation of trends in the timing of the DWFG-derived pancreas graft loss in larger studies would be expected. Second, unlike DWFG, pancreas allograft thrombosis has been discussed traditionally, particularly concerning the factors associated with graft loss. For example, factors associated with the donor, recipient, and surgery, including intraoperative hemodynamic state and organ preservation solutions, were previously reported to be risk factors for graft thrombosis^20–25^. Our results revealed that, in the presence of graft thrombus, the pancreas graft is lost very early after PTx. We also found that the donor BMI is a risk factor for graft loss due to graft thrombus. The timing of graft loss in the graft thrombus group was consistent with previous studies^22,26,27^. Our finding that the donor BMI is a risk factor for graft thrombus is consistent with previous findings^22,25,27–29^. Finally, no preoperative factors significantly associated with CR-derived graft loss were detected in SPK patients. However, more cases with CR-derived graft loss are required for the analysis than are included in this study. Considering that a solitary PTx (PAK or PTA) increased the risk of CR compared to an SPK, the case accumulation would not be easily completed.

Considering the potential clinical applications of our results, our findings will increase our understanding of the main causes of pancreas graft loss. For example, the timing of DWFG (causing 44.2% of pancreas graft loss) does not take into regard the length of the post-transplant period. In addition, increased understanding of the risk of pancreas graft loss associated with each cause may reduce delays in diagnosis and performing the post-transplant follow-up. In turn, this information may lead to improvements in the PTx outcome. For example, in patients with long-term type 1 diabetes mellitus who exhibit a high risk of DWFG (with an increasing rate of 6% per year), the clinician should closely check, before and after PTx, for systemic diseases that can cause DWFG, such as cerebro- or cardiovascular diseases, infection, or malignancy. For patients at high risk of graft thrombus, more careful postoperative management, such as more frequent checking of the blood flow by ultrasonography and administration of anti-thrombotic therapy, may be helpful for prevention. Thus, based on our results, clinicians can perform careful follow-ups, keeping in mind the specific characteristics of each cause, particularly the timing and factors associated with pancreas graft loss, which would in turn lead to earlier examinations and treatment for each cause, especially in cases at high risk for each cause. The present study results are somewhat novel in the field of PTx.

The present study has several limitations. First, the study was based on a database analysis; we searched the data for specific causes of pancreas graft loss. However, we might have missed some cases in which the pancreas graft was lost due to multiple causes. For example, in some cases, the database might have recorded pancreas graft loss due to a duodenal graft perforation, but CR may have caused the perforation. Ideally, a closer investigation of the causes is required. However, due to the retrospective study design, closer investigation was not possible. Therefore, this limitation may have affected the study results. Second, the definitions of some events, such as AR and CR, are not fixed in this database. Information regarding whether the diagnosis is based on the result of a biopsy of the pancreas graft is also not included in the database. These findings imply not only the existence of certain bias, especially among the institutions, but also room for improvement in the database. Third, this study is based on a nationwide registry in Japan, where the criteria for pancreatic graft loss is strictly determined as a return of serum C-peptide to < 0.3 ng/ml. In this context, it might be difficult to apply our results in some countries. The last limitation was the small number of SPK cases, even though the database included nationwide PTx cases. In particular, each of the three cause groups comprised low numbers of patients. If the number had been more, we could investigate factors associated with each cause of DWFG. Including more PTx cases would have enabled a closer investigation of factors that affected the outcome.

In summary, our results highlight different characteristics of the specific causes of pancreas graft loss, including the timing and associated preoperative factors after PTx. To the best of our knowledge, this study is the first to address this issue. In particular, DWFG occurred in 44.2% of the post-transplant cases, with the risk increasing at a rate of 6% per year of type 1 diabetes mellitus duration, and its timing has no regard for the length of the post-transplant period. These findings have increased our understanding of the specific causes of pancreas graft loss. Moreover, our results demonstrate the clinical utility of analyzing the specific causes of pancreas graft loss in patients undergoing PTx for type 1 diabetes mellitus.

## Data Availability

The data underlying this study cannot be shared publicly and restrictions apply to the availability of the data, which were used under license for the current study. However, data are available from the authors upon reasonable request and with permission from the Japan Society for Transplantation.
